# In Vitro and In Vivo Antimicrobial Activities of Vancomycin and Rifampin against *Elizabethkingia anophelis*

**DOI:** 10.3390/ijms242317012

**Published:** 2023-11-30

**Authors:** I-Fan Lin, Chung-Hsu Lai, Shang-Yi Lin, Ching-Chi Lee, Nan-Yao Lee, Po-Yu Liu, Chih-Hui Yang, Yi-Han Huang, Jiun-Nong Lin

**Affiliations:** 1 Division of Infectious Diseases, Department of Internal Medicine, E-Da Hospital, I-Shou University, Kaohsiung 824, Taiwan; lifeva@gmail.com (I.-F.L.);; 2 School of Medicine, College of Medicine, I-Shou University, Kaohsiung 824, Taiwan; 3 Division of Infectious Diseases, Department of Internal Medicine, Kaohsiung Medical University Hospital, Kaohsiung Medical University, Kaohsiung 807, Taiwan; amoe616@kmu.edu.tw; 4 Clinical Medicine Research Center, National Cheng Kung University Hospital, College of Medicine, National Cheng Kung University, Tainan 704, Taiwan; chichingbm85@gmail.com; 5 Division of Infectious Diseases, Department of Internal Medicine, National Cheng Kung University Hospital, Tainan 704, Taiwan; nanyao@mail.ncku.edu.tw; 6 School of Medicine, College of Medicine, National Cheng Kung University, Tainan 704, Taiwan; 7 Division of Infectious Diseases, Department of Internal Medicine, Taichung Veterans General Hospital, Taichung 407, Taiwan; pyliu@vghtc.gov.tw; 8 Department of Biological Science and Technology, Meiho University, Pingtung 912, Taiwan; 9 Department of Critical Care Medicine, E-Da Hospital, I-Shou University, Kaohsiung 824, Taiwan

**Keywords:** *Elizabethkingia anophelis*, vancomycin, rifampin, synergy, zebrafish

## Abstract

*Elizabethkingia anophelis* has emerged as a critical human pathogen, and a number of isolated reports have described the successful treatment of *Elizabethkingia* infections with vancomycin, a drug that is typically used to target Gram-positive bacteria. This study employed in vitro broth microdilution checkerboard and time-kill assays, as well as in vivo zebrafish animal models to evaluate the individual and combination antimicrobial effects of vancomycin and rifampin against *E. anophelis*. The minimum inhibitory concentration ranges of vancomycin and rifampin against 167 isolates of *E. anophelis* were 16–256 mg/L and 0.06–128 mg/L, respectively. The checkerboard assay results revealed a synergistic effect between vancomycin and rifampin in 16.8% (28/167) of the isolates. Time-kill assays were implemented for 66 isolates, and the two-drug combination had a synergistic interaction in 57 (86.4%) isolates. In vivo zebrafish studies revealed that treatment with vancomycin monotherapy, rifampin monotherapy, or vancomycin–rifampin combination therapy yielded a higher survival rate than the control group treatment with 0.9% saline. The results of this study support the use of vancomycin to treat *E. anophelis* infections.

## 1. Introduction

Bacteria of the genus *Elizabethkingia* are characterized as Gram-negative, aerobic, pale yellow-pigmented, nonmotile, incapable of fermenting glucose, non-sporulating, positive for the oxidase test, showing weak indole production, and are negative for nitrate reduction [1]. These bacteria are widely distributed in natural environments, including water, soils, fish, frogs, and insects. They can also be found in hospital tap water [1]. *Elizabethkingia* was initially described as a cause of neonatal meningitis by Elizabeth O. King, a microbiologist at the US Centers for Disease Control and Prevention, in 1959 [2]. Initially, it was named *Flavobacterium meningosepticum*. Subsequently, it was renamed as *Chryseobacterium meningosepticum* in 1994, and later reclassified as *Elizabethkingia meningoseptica* in 2005 [1]. The genus now includes seven species: *E. meningoseptica*, *E. miricola*, *E. anophelis*, *E. bruuniana*, *E. ursingii*, *E. occulta*, and *E. argenteiflava* [1,3]. Among these species, *E. anophelis*, originally isolated from the midgut of *Anopheles gambiae* mosquitoes in the Gambia, Africa by Kämpfer et al. in 2011, is associated with the highest prevalence of life-threatening infections in humans. [1,4]. Patients with *E. anophelis* infections are reported to have a mortality rate of 24–60% [1].

*Elizabethkingia* species generally exhibit multi-antibiotic resistance, including to most β-lactams, β-lactam and β-lactamase inhibitor combinations, carbapenems, aminoglycosides, and colistin; however, they have varying susceptibilities to fluoroquinolones and sulfa drugs [1]. Notably, several isolated reports have described the successful treatment of patients infected with *Elizabethkingia* using vancomycin, either as monotherapy or in combination with rifampin [5,6,7,8,9]. However, vancomycin is a glycopeptide antibiotic that is primarily active against Gram-positive bacteria; the outer membrane of Gram-negative microorganisms is composed of phospholipids and lipopolysaccharides that prevent vancomycin from penetrating to its target site [10]. Moreover, rifampin is mainly used as an adjunct in the treatment of Gram-positive bacteria because its hydrophobic characteristics impede its ability to penetrate the outer membrane of Gram-negative bacteria [11]. Therefore, it is weird that these two anti-Gram-positive bacteria agents were effective against *Elizabethkingia* species as described in the literature [5,6,7,8,9].

Unfortunately, no comprehensive study to date has investigated the in vitro and in vivo antimicrobial effects of vancomycin and rifampin against *E. anophelis*. This study employed a broth microdilution checkerboard assay, as well as a time-kill assay, to evaluate the in vitro antimicrobial effects of vancomycin monotherapy, rifampin monotherapy, and vancomycin–rifampin combination therapy against *E. anophelis*. We further examined the in vivo antimicrobial activities of these antibiotics against *E. anophelis* using a zebrafish animal model.

## 2. Results

### 2.1. Antimicrobial Susceptibility Testing

The minimum inhibitory concentrations (MICs) of vancomycin and rifampin against 167 *E. anophelis* isolates are listed in Table 1. The MIC_50_ values of vancomycin and rifampin were 32 mg/L and 0.5 mg/L, respectively. According to the Clinical and Laboratory Standards Institute guidelines breakpoint criteria of *Enterococcus faecalis*, all isolates were resistant to vancomycin, but 95.2% were susceptible to rifampin.

### 2.2. Synergistic Effects Using Broth Microdilution Checkerboard Assays

**Figure 1:**
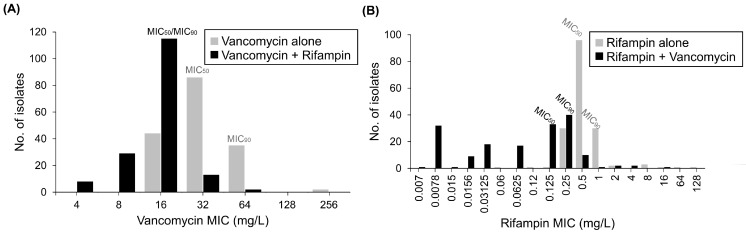
Minimum inhibitory concentrations (MICs) of vancomycin (**A**) and rifampin (**B**) against 167 isolates of *E. anophelis* using the broth microdilution checkerboard assays. (**A**) After addition of rifampin, the MIC_50_ and MIC_90_ of vancomycin decrease. (**B**) When combination with vancomycin, the MIC_50_ of rifampin reduced. MIC_50_, MIC at which 50% of the isolates tested are inhibited. MIC_90_, MIC at which 90% of the isolates tested are inhibited.

After the addition of rifampin, the MIC_50_ and MIC_90_ of vancomycin decreased from 32 mg/L to 16 mg/L, and from 64 mg/L to 16 mg/L, respectively (Table 1 and Figure 1A). When combined with vancomycin, the MIC_50_ of rifampin decreased from 0.5 mg/L to 0.125 mg/L, and the MIC_90_ decreased from 1 mg/L to 0.25 mg/L (Table 1 and Figure 1B). Synergistic effects were identified in 16.8% (28/167) of isolates, and no antagonistic effects were noted between vancomycin and rifampin (Table 2).

### 2.3. Antimicrobial Effects Using Time-Kill Assays

**Figure 2:**
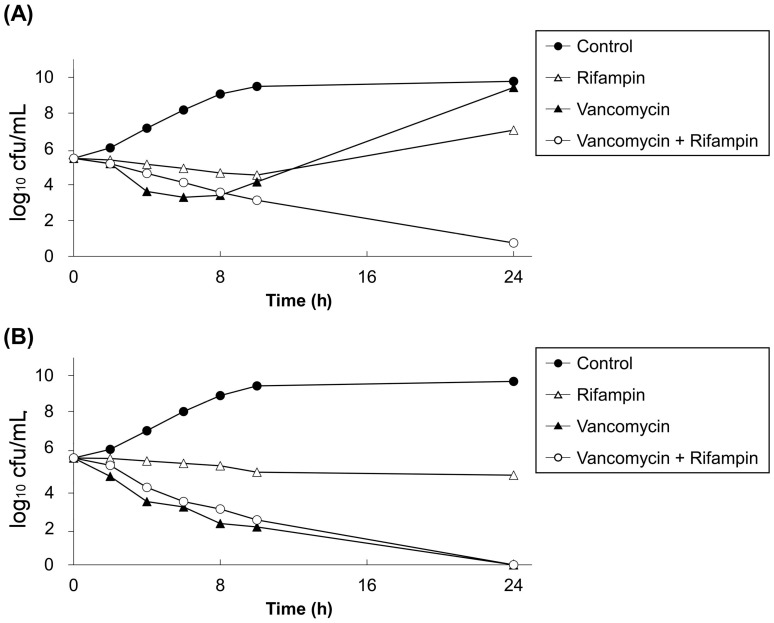
Time-kill assays for vancomycin and rifampin, alone and in combination, against 66 isolates of *E. anophelis*. (**A**) Isolates presenting with synergistic interaction of vancomycin and rifampin (*n* = 57). (**B**) Isolates presenting with indifferent synergistic interaction of vancomycin and rifampin (*n* = 9). The concentrations of vancomycin and rifampin were 1 × MIC. Data points represent the median bacterial colony counts of isolates.

The time-kill assays results revealed a synergistic interaction between vancomycin and rifampin against *E. anophelis* in 86.4% (57/66) of isolates, as well as no antagonistic interactions (Table 2). The agreement rate of synergy between the broth microdilution checkerboard assay and time-kill assays was 22.7%. The median bacterial colony counts of the isolates for vancomycin and rifampin in time-kill assays are presented in Figure 2. For the isolates with the synergistic effects of vancomycin and rifampin (Figure 2A), neither vancomycin nor rifampin met the criteria for a bactericidal effect, but vancomycin monotherapy exhibited a better killing effect than rifampin monotherapy within the first 8 h. Synergistic and bactericidal effects were observed for vancomycin and rifampin combination therapy after 14 h. For those manifesting indifferent interactions between vancomycin and rifampin (Figure 2B), rifampin exhibited only a bacteriostatic effect, but vancomycin exhibited a bactericidal effect, which was almost parallel to that of vancomycin plus rifampin in the time-kill curve. According to the synergy criteria, no synergistic interaction occurred between vancomycin and rifampin because the bactericidal ability of vancomycin was similar to that of vancomycin–rifampin combination therapy.

### 2.4. Antimicrobial Activities in Zebrafish Infected with E. anophelis

**Figure 3:**
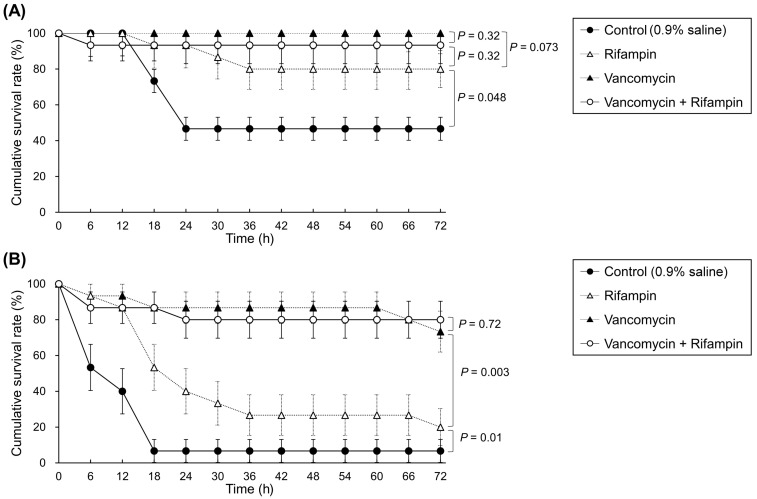
Kaplan–Meier survival curves of the 72-h cumulative survival rate for zebrafish infected with *E. anophelis*. (**A**) Strain ED204-47. The log rank tests: rifampin vs. control: *p* = 0.048; vancomycin vs. control, *p* = 0.001; vancomycin-rifampin combination vs. control, *p* = 0.01. There was no statistically significant difference in the survival rates of zebrafish treated with vancomycin alone, rifampin alone, and vancomycin-rifampin combination. (**B**) Strain ED550-81. The log rank tests: rifampin vs. control, *p* = 0.01; vancomycin vs. control, *p* < 0.001; vancomycin-rifampin combination vs. control, *p* < 0.001; vancomycin vs. vancomycin-rifampin combination, *p* = 0.72; rifampin vs. vancomycin, *p* = 0.003; rifampin vs. vancomycin-rifampin combination, *p* = 0.003. The symbols and vertical bars indicate average and standard errors of cumulative survival rates, respectively. Strain ED204-47: vancomycin MIC = 16 mg/L and rifampin MIC = 8 mg/L. Strain ED550-81: vancomycin MIC = 64 mg/L and rifampin MIC = 8 mg/L. Each study group comprised five zebrafish, and the experiment was repeated twice. Intraperitoneal antibiotic injections were administered 2 h after bacterial injection. Dosages: vancomycin, 15 mg/kg every 12 h for 72 h after a loading dose of 25 mg/kg; rifampin, 5 mg/kg every 12 h for 72 h.

After the intraperitoneal administration of *E. anophelis*, zebrafish infected with strain ED550-81 had a higher mortality rate than those infected with strain ED204-47 (93.3% vs. 53.3%; *p* < 0.001; Figure 3). For zebrafish infected with the less virulent strain of ED204-47, the 72-h survival rates of those treated with vancomycin monotherapy, rifampin monotherapy, and vancomycin plus rifampin combination therapy were 100%, 80%, and 93.3%, respectively. Compared with those treated with 0.9% saline, zebrafish receiving either rifampin monotherapy (*p* = 0.048), vancomycin monotherapy (*p* = 0.001), or vancomycin plus rifampin combination therapy (*p* = 0.01) had significantly higher survival rates. The results indicated no significant difference in the survival rates of zebrafish treated with vancomycin monotherapy, rifampin monotherapy, and vancomycin–rifampin combination therapy (Figure 3A). For zebrafish infected with the more virulent strain of ED550-81, the 72-h survival rate was the highest for those treated with vancomycin–rifampin combination therapy (80%), followed by those treated with vancomycin monotherapy (73.3%) and rifampin monotherapy (20%; Figure 3B). Zebrafish receiving rifampin monotherapy (*p* = 0.01), vancomycin monotherapy (*p* < 0.001), or vancomycin plus rifampin (*p* < 0.001) combination therapy had significantly higher survival rates than those treated with 0.9% saline. The results revealed no significant difference in the survival rates of zebrafish treated with vancomycin monotherapy and those treated with vancomycin–rifampin combination therapy (*p* = 0.72). However, the zebrafish infected with strain ED550-81 that received rifampin monotherapy had a significantly lower survival rate than those that received vancomycin monotherapy (*p* = 0.003), or vancomycin plus rifampin combination therapy (*p* = 0.003).

## 3. Discussion

As previously mentioned, several pediatric patients with *E. meningoseptica* infections have been effectively treated with vancomycin, either alone or in combination with rifampin [5,6,7,8,9]. For example, Di Pentima et al. [6] documented the cases of four neonates who had community-acquired *E. meningoseptica* infections, including three with meningitis and one with pneumonia. Among these three neonates with meningitis, treatment with either vancomycin plus rifampin, or trimethoprim-sulfamethoxazole plus rifampin, resulted in their survival without subsequent neurological deficits or hydrocephalus. The newborn with pneumonia developed *E. meningoseptica* bacteremia, and his condition improved following a 10-day course of therapy involving ceftazidime and vancomycin. Unfortunately, this infant later acquired *Staphylococcus aureus* bacteremia, and tragically passed away at the age of two months. Ceyhan et al. [9] reported three outbreaks of *E. meningoseptica* infections that affected a total of 13 pediatric patients, with an overall mortality rate of 30.7%, in Turkey. The age of the patients varied from four days to 23 months old. The clinical symptoms observed included meningitis, pneumonia, bacteremia, cellulitis, fasciitis, and sepsis. *E. meningoseptica* was detected in the blood of 12 patients, and in the cerebrospinal fluid of four patients. Out of the 13 patients, ten were treated with vancomycin. Among these, all three patients who received a combination therapy of vancomycin and rifampin survived. In contrast, three out of the seven patients who received vancomycin monotherapy did not survive [9].

In addition to pediatric patients, vancomycin has also proven to be an effective treatment for non-neonatal patients with *E. meningoseptica* infections. Jean et al. [12] conducted a literature review, and analyzed 12 patients with *E. meningoseptica* bacteremia who received vancomycin-inclusive therapy. Among these patients, six had end-stage renal disease, five had immunocompromising factors other than end-stage renal disease, and only one was immunocompetent. Only one patient, who had diabetic nephropathy, end-stage renal disease, and lung cancer, unfortunately died after receiving vancomycin treatment. The overall mortality rate for these *E. meningoseptica* bacteremic patients who received vancomycin-inclusive therapy was 8.3% [12].

As revealed in our previous investigation, as well as in other relevant studies [4,13,14], *E. anophelis* accounts for approximately 59.3–98.7% of *Elizabethkingia* infections in humans. Han et al. [13] conducted an investigation involving 86 *Elizabethkingia* isolates collected in South Korea between 2009 and 2015. They used 16S ribosomal ribonucleic acid (rRNA) gene sequencing, and found that 17 isolates (19.8%) were *E. meningoseptica*, 18 isolates (20.9%) were *E. miricola*, and 51 isolates (59.3%) were *E. anophelis*. Similarly, Chew et al. [14] analyzed the species of 79 *Elizabethkingia* isolates collected in Singapore from 2009 to 2017, also using 16S rRNA gene sequencing. Their findings revealed that 78 out of 79 isolates (98.7%) were *E. anophelis*, while one isolate (1.3%) was identified as *E. meningoseptica*. Moreover, almost all *E. anophelis* isolates are mistakenly identified as *E. meningoseptica* when using traditional biochemical-based phenotyping methods or matrix-assisted laser desorption ionization–time of flight mass spectrometry (MALDI-TOF MS) [15]. In our previous study, we compared four commonly used automated microbiology systems in clinical microbial laboratories to 16S rRNA gene sequencing for the identification of *Elizabethkingia* species. The results showed that only 24.5% of the isolates were correctly identified when using the API/ID32 phenotyping kits (bioMérieux, Marcy l’Etoile, France) and the Phoenix 100 ID/AST automated microbiology system (Becton, Dickinson Co., Sparks, MD, USA). Furthermore, 28.9% of the isolates were correctly identified using the Vitek 2 automated identification system (bioMérieux) and the Vitek MALDI-TOF MS system (bioMérieux). Notably, none of the isolates of *E. anophelis* were accurately identified using these four microbial identification systems [15]. Therefore, the majority of *E. meningoseptica*-infected patients who were successfully treated with vancomycin in the old literature [5,6,7,8,9], could actually have had their illness caused by *E. anophelis*.

Studies have described that vancomycin has a high rate of susceptibility against *Elizabethkingia* when using in vitro disk diffusion testing, and those patients therefore received ‘appropriate’ antibiotic treatment with vancomycin [7,8,9]. For example, the 13 isolates of *E. meningoseptica* obtained from pediatric patients, as reported by Ceyhan et al. [9], were found to be susceptible to vancomycin when subjected to disk diffusion testing. Therefore, these patients received the treatment of vancomycin. Nevertheless, using the Clinical and Laboratory Standards Institute-recommended standard methods of agar dilution or broth microdilution, more recent investigations have revealed that most *Elizabethkingia* species exhibit high MICs to vancomycin [6,14]. The present study also revealed high MICs to vancomycin in the *E. anophelis* isolates collected from multiple hospitals in Taiwan (MIC_50_ = 32 mg/L; MIC_90_ = 64 mg/L). The disk diffusion method has been found to overestimate the susceptibility rate of vancomycin against *Elizabethkingia* [16]. As a results, high vancomycin MICs were inferred in the *Elizabethkingia* isolates which MICs were determined using disk diffusion testing in the literature [5,7,8,9].

A gold-standard method for determining synergistic interactions has not been established. The outcomes obtained through various methods exhibit considerable variability, and are typically contingent on the specific microorganisms under examination [17]. The checkerboard assay is widely employed for assessing the synergistic effects of different agents due to its rapid execution and its ability to provide MIC ranges [18]. The time-kill assay can provide data on bactericidal and bacteriostatic activity, and information about the pharmacokinetic–pharmacodynamic relationship of the compound [17,19]. Therefore, the time-kill assay is often regarded as one of the most accurate methods for implementing synergy testing between antimicrobial agents; however, this technique is time-consuming and labor-intensive. As mentioned, there was a huge discrepancy of the synergy results between vancomycin and rifampin determined using checkerboard and time-kill assays in this study. Synergy was observed in 16.8% of *E. anophelis* isolates when using broth microdilution checkerboard assays, whereas synergy was observed in 86.4% of isolates when using time-kill assays. Although the time-kill assay is more complex than the checkerboard assay, this study recommends using this technique for the synergistic evaluation of antimicrobial agents against *Elizabethkingia*.

In addition to the in vitro broth microdilution checkerboard and time-kill assays, this study employed an in vivo zebrafish animal model to examine antimicrobial effects. The results revealed no synergistic interaction between vancomycin and rifampin combination therapy in zebrafish infected with two separate strains of *E. anophelis*. Like the excellent killing effect of vancomycin alone resulting no synergistic effect between these two agents in the time-kill assays, single therapy with vancomycin alone provided an excellent survival rate of 93.3% (strain ED204-47 infection) and 73.3% (strain ED550-81 infection). This resulted in no statistical difference in synergistic interaction between vancomycin and rifampin in this in vivo animal study. However, rifampin has the merits of potent bactericidal activity, high intracellular concentration, and favorable biofilm penetrating activity [11]. The results from both the time-kill assays and the animal model study suggest that the need for combining vancomycin and rifampin in the treatment of *E. anophelis* infections hinges on whether vancomycin alone is effective.

Vancomycin acts through binding to the D-Ala-D-Ala terminus of peptidoglycan precursors, therefore preventing cell wall synthesis [10]. Rifampin, which acts through inhibiting DNA-dependent RNA polymerase, is generally used in combination with another active antibiotic because rifampin monotherapy induces the development of rapid antibiotic resistance [11]. Both vancomycin and rifampin are regarded as effective against only Gram-positive bacteria because the outer membrane of Gram-negative bacteria impedes the penetrating ability of these drugs to their target sites [10,11]. However, increasing the permeability of the outer membrane could enhance the effectiveness of anti–Gram-positive antibiotics against Gram-negative bacteria [11,20]. One study observed that vancomycin conjugated with arginine had greater outer membrane penetration and enhanced activity against Gram-negative bacteria [20]. Another study revealed that polymyxins could effectively permeabilize the outer membrane of Gram-negative bacteria and increase the effectiveness of rifampin against these pathogens [11]. The present study revealed that vancomycin and rifampin demonstrated antimicrobial effects against *E. anophelis*. Theoretically, this Gram-negative pathogen should be inherently resistant to vancomycin and rifampin. However, our results demonstrated a strong antimicrobial effect of vancomycin against *E. anophelis*, both as a monotherapy and in combination with rifampin. The present study did not add any compound that could influence or change the outer membrane of *E. anophelis*. Moreover, research has not described the structure of the outer membrane of *E. anophelis* as being different from that of other Gram-negative bacteria. The reason for the antimicrobial effects of vancomycin and rifampin against *E. anophelis* remains undetermined.

## 4. Material and Methods

### 4.1. Bacterial Strains

*Elizabethkingia* isolates had been routinely collected from patients between 2005 and 2020, in accordance with clinical requirements in five hospitals in Taiwan, including E-Da Hospital, Kaohsiung Medical University Hospital, E-Da Cancer Hospital, National Cheng Kung University Hospital, and Taichung Veterans General Hospital. The isolates were initially identified as *Elizabethkingia* species in clinical microbiology laboratories through the use of traditional biochemical-based microbial identification systems, or MALDI-TOF MS, including using API/ID32 phenotyping kits, the Phoenix 100 ID/AST automated microbiology system, the Vitek 2 automated identification system, or the Vitek MALDI-TOF MS system. These isolates were stored as glycerol stocks at −80 °C until use. Finally, a total of 167 *E. anophelis* isolates were employed in this study. 

### 4.2. Bacterial DNA Extraction

The genomic deoxyribonucleic acid (DNA) was extracted using the Wizard Genomic DNA Purification Kit (Promega, Madison, WI, USA). Thawed bacteria were subcultured on blood agar plates at 37 °C for 18 to 24 h, and then transferred to 1.5 mL microcentrifuge tubes. After centrifugation at 13,000–16,000× *g* for 2 min, the supernatant fluid was removed. The cells were resuspended in 600 µL of Nuclei Lysis Solution and incubated at 80 °C for 5 min to lyse the cells. RNase Solution (3 µL) was added to the cell lysate and incubated at 37 °C for 15 to 60 min. Protein Precipitation Solution (200 µL) was added to the RNase-treated cell lysate and vigorously vortexed for 20 s to mix the solution with the cell lysate. The supernatant of the bacterial solution, after centrifugation at 13,000–16,000× *g* for 3 min, was transferred to a clean 1.5 mL microcentrifuge tube containing 600 µL of room temperature isopropanol. The fluid was gently mixed until the DNA formed visible thread-like strands and became a visible mass. The supernatant, after another centrifugation at 13,000–16,000× *g* for 2 min, was discarded, and 600 µL of 70% ethanol was added to wash the DNA pellet. The DNA pellet was then transferred to clean absorbent paper and allowed to air-dry for 10 to 15 min. To rehydrate the DNA, DNA Rehydration Solution (100 µL) was added, and the mixture was incubated at 65 °C for 1 h. The rehydrated DNA was then stored at 2–8 °C.

### 4.3. 16S rRNA Gene Sequencing

Accurate species of *Elizabethkingia* were identified using 16S rRNA gene sequencing [4]. The primers of polymerase chain reaction (PCR) for the 16S rRNA sequence were: 8f (5′-CACGGATCCAGACTTTGATYMTGGCTCAG-3′) and 1512r (5′-GTGAAGCTTACGGYTAGCTTGTTACGACTT-3′). The amplification of the 1498-bp 16S rRNA was performed using the Simpli Amp Thermal Cycler (Applied biosystems by Thermo Fisher Scientific, Waltham, MA, USA). The conditions used for 16S rRNA amplification were an initial extended denaturation step of 94 °C for 5 min, followed by 35 cycles of 10 s at 94 °C, 20 s at 54 °C, 2 min at 68 °C, and a final 5 min at 68 °C. The PCR reactions (50 µL) contained EconoTaq PLUS GREEN 2X Master Mix (Lucigen) (25 µL), 8f primer (25 pmole) (1 µL), 1512r primer (25 pmole) (1 µL), genomic DNA (1 µL), and ddH_2_O (22 µL). Amplification products were confirmed by 1% agarose gel electrophoresis. The sequences of the amplicons were performed at a biotechnology company (Genomics BioSci & Tech Corp., Taipei, Taiwan) using the Applied Biosystems 3730xl DNA Analyzer. The primers for sequencing were: 8f (5′-GGATCCAGACTTTGATYMTGGCTCAG-3′), 534r (5′-ATTACCGCGGCTGCTGG-3′), 534f (5′-CCAGCAGCCGCGGTAAT-3′), and 968f (5′-AACGCGAAGAACCTTAC-3′).

### 4.4. MIC Determination

The MICs of vancomycin and rifampin (Sigma-Aldrich, St. Louis, MO, USA) against *E. anophelis* were determined using 96-well broth microdilution panels, in accordance with the manufacturer’s instructions (Thermo Fisher Scientific, Oakwood Village, OH, USA). Briefly, three to five colonies of isolates picked up from agar plates were mixed in sterile water, and then were adjusted to a 0.5 McFarland Standard. Then, a 10 µL bacterial solution was mixed with 11 mL of cation-adjusted Mueller–Hinton broth containing TES buffer (2-{[1,3-Dihydroxy-2-(hydroxymethyl)-2-propanyl] amino} ethanesulfonic acid). Subsequently, 50 µL of this broth suspension was placed in a 96-well plate and incubated at a temperature between 34 and 36 °C in a non-CO_2_ incubator. After 18 to 24 h of incubation, the results were examined, and the MIC was determined as the lowest concentration of antibiotics that prevented visible bacterial growth.

### 4.5. Susceptibility Breakpoints

The breakpoints for susceptibility testing were determined based on the interpretive standards for *Enterococcus faecalis* because the Clinical and Laboratory Standards Institute do not provide interpretive criteria for these antibiotics against “other non-Enterobacteriaceae” [21]. A MIC of ≤4 mg/L indicated susceptibility to vancomycin, whereas MICs of 8–16 mg/L and ≥32 mg/L indicated intermediate sensitivity and resistance, respectively. For rifampin, MICs of ≤1, 2, and ≥4 mg/L indicated susceptibility, intermediate sensitivity, and resistance, respectively.

### 4.6. Checkerboard Assays

To analyze the synergistic effect of vancomycin and rifampin combination therapy, this study implemented a two-agent broth microdilution checkerboard assay for all 167 *E. anophelis* isolates, as described in a relevant study [18]. Bacterial solutions with a 0.5 McFarland standard were added to a 96-well plate with serial two-folded dilutions of vancomycin (4–256 mg/L) and rifampin (0.007–8 mg/L). Each 100-µL well ultimately contained approximately 5 × 10^5^ colony-forming unit (cfu)/mL. MICs were then determined. The fractional inhibitory concentrations (FICs) were calculated as follows: FIC of vancomycin (FIC_VA_) = MIC of vancomycin in combination therapy/MIC of vancomycin monotherapy; FIC of rifampin (FIC_RIF_) = MIC of rifampin in combination therapy/MIC of rifampin monotherapy; and FIC index = FIC_VA_ + FIC_RIF_. The combination was defined as synergistic when the FIC index was ≤0.5, indifferent when the FIC index was >0.5 to ≤4, and antagonistic when the FIC index was >4 [18].

### 4.7. Time-Kill Assay

In this study, a total of 66 isolates from different strains were randomly chosen for the time-kill assay because of their time-consuming, labor-intensive, and costly nature. The bacterial strains were cultured overnight at a temperature of 35 °C in cation-adjusted Mueller–Hinton broth. The bacterial suspension was then adjusted to a 1.7 McFarland standard, which corresponds to approximately 5 × 10^8^ cfu/mL. Subsequently, 25 µL of the adjusted bacterial suspension was introduced into a flask containing 25 mL of cation-adjusted Mueller–Hinton broth, resulting in a bacterial concentration of approximately 5 × 10^5^ cfu/mL. Solutions of vancomycin and rifampin, each at a concentration equivalent to 1 × MIC, were added to these bacterial suspensions. After an incubation time of 0, 2, 4, 6, 8, 10, and 24 h, 50-µL aliquots of the bacterial suspensions were added to 450 µL of phosphate-buffered saline. The colony number was counted on Mueller–Hinton agar plates inoculated with 100-µL aliquots of 10-fold serially-diluted bacterial suspension after 20–22 h of incubation at 37 °C. Antibiotics were classified as bactericidal if the number of colonies decreased ≥ 3 log_10_ within 24 h compared with the initial inoculum [22]. Synergy between the combination of vancomycin and rifampin was defined as a ≥2 log_10_ decrease in colony count compared with the most active single agent. Antagonism was defined as a ≥2 log_10_ increase in colony count compared with the most single active agent. Indifference was defined as the change in colony count between synergy and antagonism [18].

### 4.8. Zebrafish Housing and Antimicrobial Study

The animal study followed the guidelines for the care and use of laboratory animals, and was approved by the Institutional Animal Care and Use Committee of E-Da Hospital (IACUC-EDAH-110028). For this study, we utilized approximately 7-month-old wild-type zebrafish (AB strain) that were acquired from a commercial distributor in Taiwan (G. Fish Animal Model Co., Taipei, Taiwan). To acclimate the zebrafish to the laboratory environment, all experiments were conducted at least one week after their arrival at our laboratory. The zebrafish were accommodated in multiple 1.5-L tanks and subjected to a 14-h light/10-h dark cycle at a temperature of 28 °C. They were provided with a diet containing crude fiber (maximum 2.5%), crude moisture (maximum 6.32%), crude fat (minimum 6.8%), and crude protein (minimum 46.8%), along with essential vitamins such as vitamin A, vitamin B1-3, vitamin B6, vitamin D3, vitamin E, vitamin K3, and calcium pantothenate. These zebrafish were fed twice daily. Two *E. anophelis* strains with high MICs of vancomycin and rifampin (strain ED204-47 with vancomycin MIC of 16 mg/L and rifampin MIC of 8 mg/L; and strain ED550-81 with vancomycin MIC of 64 mg/L and rifampin MIC of 8 mg/L) were arbitrarily chosen from the collection of the 167 isolates for the evaluation of antimicrobial effects using the zebrafish animal model. A 10-µL bacterial solution (7.5 × 10^9^ cfu/mL) was injected into the peritoneal cavity of zebrafish using a 35G beveled steel needle. Intraperitoneal antibiotic injections were administered 2 h after bacterial injection. The following dosages of vancomycin and rifampin were administered: vancomycin, 15 mg/kg every 12 h for 72 h after a loading dose of 25 mg/kg; and rifampin, 5 mg/kg every 12 h for 72 h. Each study group comprised five zebrafish, and the experiment was repeated twice. Kaplan–Meier survival analysis, and the log-rank test were implemented using SPSS version 19.0 (IBM, 2010, Armonk, NY, USA). Statistical significance was indicated at a two-tailed *p* value of <0.05. 

## 5. Conclusions

*E. anophelis* has emerged as a critical pathogen that causes life-threatening infections in patients. Therapeutic options for this multidrug-resistant microorganism are limited. This study revealed the excellent antimicrobial efficacy of vancomycin against *E. anophelis*, either as a monotherapy or in combination with rifampin. Further clinical trials are warranted to evaluate the efficacy and outcomes of vancomycin monotherapy and vancomycin–rifampin combination therapy for *E. anophelis* infections in humans.

## Figures and Tables

**Table 1:** Antimicrobial susceptibility testing of *E. anophelis* isolates.

Antimicrobial Agent	MIC (mg/L)			Interpretation of Susceptibility, *n* (%) ^d^		
	Range	MIC_50_^b^	MIC_90_^c^	Susceptible	Intermediate	Resistant
Vancomycin						
Alone	16–256	32	64	0	44 (26.3)	123 (73.7)
+rifampin ^a^	4–64	16	16	8 (4.8)	144 (86.2)	15 (9)
Rifampin						
Alone	0.06–128	0.5	1	159 (95.2)	2 (1.2)	6 (3.6)
+vancomycin ^a^	0.078–128	0.125	0.25	162 (97)	2 (1.2)	3 (1.8)

^a^ Determined using broth microdilution checkerboard assay. ^b^ MIC at which 50% of the isolates tested are inhibited. ^c^ MIC at which 90% of the isolates tested are inhibited. ^d^ For vancomycin, MIC ≤ 4 mg/L is interpreted as susceptible; MIC 8−16 mg/L, intermediate; ≥32 mg/L, resistant. For rifampin, MIC ≤ 1 mg/L is interpreted as susceptible; MIC 2 mg/L, intermediate; ≥4 mg/L, resistant.

**Table 2:** Fractional inhibitory concentration (FIC) indices using checkerboard and time-kill assays for *E. anophelis*.

Synergy Test	Interpretation	FIC index		FIC_VA_^a^		FIC_RIF_^b^	
		Range	Median (IQR)	Range	Median (IQR)	Range	Median (IQR)
Checkerboard assay (*n* = 167)	Synergy (*n* = 28; 16.8%)	0.266−0.5	0.50 (0.13)	0.25	0.25 (0)	0.016−0.25	0.25 (0.13)
	Indifference (*n* = 139; 83.2%)	0.508−1.5	0.75 (0.44)	0.125−1.0	0.50 (0)	0.001−0.5	0.25 (0.47)
	Antagonism (*n* = 0)	-	-	-	-	-	-
Time-kill assay (*n* = 66)	Synergy (*n* = 57; 86.4%)	-	-	-	-	-	-
	Indifference (*n* = 9; 13.6%)	-	-	-	-	-	-
	Antagonism (*n* = 0)	-	-	-	-	-	-

^a^ FIC of vancomycin (FIC_VA_) = MIC of vancomycin in combination/MIC of vancomycin alone. ^b^ FIC of rifampin (FIC_RIF_) = MIC of rifampin in combination/MIC of rifampin alone. IQR, interquartile range; MIC, minimum inhibitory concentration.

## Data Availability

All data generated or analyzed during this study are included in this published article.
